# Preditores de Fibrilação Atrial no Monitoramento de Holter após Acidente Vascular Cerebral - Um Flashback de Dez Anos

**DOI:** 10.36660/abc.20210660

**Published:** 2022-07-29

**Authors:** Tânia Proença, Ricardo Alves Pinto, Miguel Martins de Carvalho, Carla Sousa, Paula Dias, Manuel Campelo, Filipe Macedo

**Affiliations:** 1 Universitário de São João Centro Hospitalar Porto Portugal Centro Hospitalar Universitário de São João, Porto – Portugal; 2 Universidade do Porto Faculdade de Medicina Porto Portugal Universidade do Porto Faculdade de Medicina, Porto – Portugal

**Keywords:** Fibrilação Atrial, Acidente Vascular Cerebral (AVC)/terapia, Taquicardia Supraventricular, Tromboembolismo/terapia, Fatores de Risco, Eletrocardiografia, Ambulatorial/métodos

## Introdução

A fibrilação atrial (FA) é um importante fator de risco para eventos tromboembólicos, aumentando cinco vezes o risco de acidente vascular cerebral; também está associada a eventos mais graves e a um risco maior de recorrência de AVC.^1,2^ Por outro lado, o diagnóstico de FA como causa de acidente vascular cerebral isquêmico altera a abordagem terapêutica com grande impacto prognóstico.^3,4^ A detecção de FA previamente desconhecida após o AVC é crucial, e vários estudos estabeleceram a eficácia do monitoramento de ECG para a detecção de FA pós-AVC.^1^ De acordo com as Diretrizes do ESO para o manejo de acidente vascular cerebral isquêmico e ataque isquêmico transitório (AIT), após a fase aguda, deve ser realizada um monitoramento Holter ECG de 24 horas.^5^ As novas diretrizes da ESC recomendam o registro de ECG de curto prazo pelo menos nas primeiras 24 h e monitoramento contínuo de ECG por pelo menos 72 h sempre que possível no AVC criptogênico.^1^

## Métodos

Realizamos um estudo retrospectivo em um único centro terciário em pacientes que sofreram AVC isquêmico ou AIT e realizamos Holter entre outubro de 2009 e outubro de 2011. Todos os pacientes consecutivos foram selecionados e aqueles com FA ou FA prévia foram excluídos. Acompanhamos esses pacientes por 8 a 10 anos, observamos a incidência de FA e avaliamos os preditores clínicos, eletrocardiográficos e ecocardiográficos de FA de início recente.

Atividade ectópica supraventricular excessiva (AESVE) foi definida como ≥ 500 contrações atriais prematuras por 24 horas ou qualquer episódio de taquicardia supraventricular sustentada.^6^

A análise estatística foi realizada no IBM SPSS Statistics versão 25. As variáveis categóricas foram comparadas pelo teste do chi-quadrado, e as diferenças foram consideradas estatisticamente significativas quando o valor de p < 0,05.

## Resultados

No total, foram incluídos 104 pacientes; 79,5% tiveram AVC e 20,5% tiveram AIT; 45,7% eram do sexo feminino; a média de idade foi de 63,8 ± 14,7 anos na época do evento (tabela 1). Em relação aos fatores de risco cardiovascular, T 59,0% apresentavam hipertensão, 47,4% dislipidemia, 19,5% diabetes, 43,6% fumantes ou ex-fumantes e 66,7% etilistas. Em relação às características ecocardiográficas, 98% dos pacientes apresentaram fração de ejeção sistólica normal e apenas 2% apresentaram fração de ejeção discretamente alterada; o diâmetro médio do átrio anteroposterior esquerdo era de 39 mm e 60% dos pacientes apresentavam insuficiência mitral não significativa. Holter de 24 horas revelou AESVE em 13,5% dos pacientes e FA paroxística em 1,9%. Todos os pacientes com FA paroxística detectada no Holter tiveram AVC e tinham mais de 55 anos.

**Tabela 1:** Características da linha de base e resultados do acompanhamento

N		104
Idade, anos (IQR)		63,8 (49,1-78,5)
Mulher, %		45,7
Hipertensão, %		59,0
Dislipidemia, %		47,4
Diabetes, %		19,5
Fumante ou ex-fumante, %		43,6
Grandes consumidores de álcool, %		66,7
Diâmetro médio do átrio esquerdo, mm		39
Função ventricular sistólica esquerda, %		
	• Normal	98,0
	• Levemente reduzido	2,0
Evento agudo, %		
	• Derrame	79,5
	• AIT	20,5
Resultados de Holter na linha de base		
	• FA, %	1,9
	• AESVE, %	13,5
No seguimento		
	• FA, %	11,5

AIT: ataque isquêmico transitório; FA: fibrilação atrial; AESVE: atividade ectópica supraventricular excessiva.

**Figura 1:**
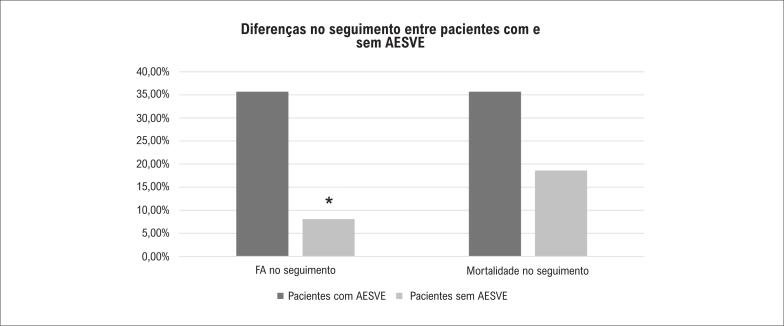
Diferenças no seguimento entre pacientes com e sem AESVE. A AESVE na apresentação foi significativamente associada à FA de início recente no seguimento (35,7% vs. 8,1%, p=0,003) e parece estar relacionada a maior mortalidade (35,7% vs 18,6% p=0,145). AESVE: atividade ectópica supraventricular excessiva; FA: fibrilação atrial.

Em um seguimento de 8-10 anos, a FA de início recente foi detectada em 11,5% dos pacientes; estes tiveram mortalidade semelhante aos em ritmo sinusal sustentado (16,7% vs. 21,1%, p=0,724). A ingestão de álcool, fator de risco estabelecido para o desenvolvimento de FA, foi associada a um aumento não significativo da FA (18,0% vs. 11,5%, p=0,464), enquanto fatores de risco cardiovascular, aumento do átrio esquerdo, insuficiência mitral não foram associados com desenvolvimento de FA. Em relação às contrações atriais prematuras (CAPs), a documentação de AESVE na apresentação mostrou-se significativamente associada ao novo início de FA no seguimento (35,7% vs. 8,1%, p=0,003). A AESVE também parece estar relacionada à maior mortalidade no seguimento de longo prazo, embora essa diferença não tenha sido estatisticamente significativa (35,7% vs. 18,6%, p=0,145) (Figura 1).

## Conclusão

Nosso estudo corrobora relatos anteriores sugerindo que CAPs excessivos aumentaram o risco de morte e FA.^6,8^ O Copenhagen Holter Study mostrou que CAPs excessivos foram associados a um risco aumentado de morte, acidente vascular cerebral e internações por FA em um acompanhamento médio de 6,3 anos.^7^ A mesma coorte foi acompanhada por 15 anos, e os pacientes com ≥ 30 CAPs/hora ou com qualquer execução de ≥ 20 CAPs tiveram um risco aumentado de AVC isquêmico além de desenvolver FA. Nesse estudo, o AVC foi mais frequentemente a primeira apresentação clínica do que a FA.^8^ Todos esses relatos e o presente estudo lançaram a questão se os pacientes com ESVEA se beneficiam da anticoagulação. De facto, a atribuição da fibrilação auricular como causa do AVC altera totalmente a terapêutica do doente, pelo que é da maior importância não só identificar os doentes com FA paroxística, mas também reconhecer quais são os que têm mais risco de ter episódios ocultos de FA. O Holter de 24 horas permite a detecção de FA paroxística, mas aparentemente com baixa eficácia. Em nosso estudo, apenas 1,9% dos pacientes foram identificados com FA imediatamente após AVC ou AIT, enquanto 11,5% apresentaram FA no seguimento de longo prazo.

Nosso estudo mostrou que a AESVE é um forte preditor de FA de início recente, destacando a importância do monitoramento de ECG. Esse achado, combinado com outros fatores de risco, como acidente vascular cerebral embólico de origem desconhecida, pode ser usado para identificar pacientes com maior risco de desenvolver FA que se beneficiam de um monitoramento de ECG de longo prazo ou de um acompanhamento mais regular.
