# Supplementary material for: Preditores de Fibrilação Atrial no Monitoramento de Holter após Acidente Vascular Cerebral - Um Flashback de Dez Anos
Source: Arq Bras Cardiol. 2022 Jul 29;119(2):346–8. [Article in Portuguese] doi: 10.36660/abc.20210660 (PMC9363065; doi:10.36660/abc.20210660)
Supplement: Translation

# Translation: Predictors of Atrial Fibrillation in Holter Monitoring after Stroke – A Ten Year Flashback

**Authors:** Tânia Proença, Ricardo Alves Pinto, Miguel Martins de Carvalho, Carla Sousa, Paula Dias, Manuel Campelo, Filipe Macedo

**Affiliations:** 1 Porto Portugal Centro Hospitalar Universitário de São João, Porto – Portugal; 2 Porto Portugal Universidade do Porto Faculdade de Medicina, Porto – Portugal

**Keywords:** Atrial Fibrillation, Stroke/therapy, Tachycardia, Ectopic Atrial, Thromboembolis/therapy, Risk Factors, Electrocardiography, Ambulatory/methods

## Introduction

Atrial fibrillation (AF) is a major risk factor for thromboembolic events, increasing five times the risk of stroke; it is also associated with more severe events and a higher risk of stroke recurrence. ^1,2^ On the other hand, the diagnosis of AF as the cause of ischaemic stroke shifts the therapeutic approach with a major prognostic impact.^3,4^ Detection of previously unknown AF after stroke is crucial, and several studies have established the effectiveness of ECG monitoring for post-stroke AF detection.^1^ According to ESO Guidelines for managing ischaemic stroke and transient ischaemic attack (TIA), after the acute phase, a 24-hour Holter ECG monitoring should be performed.^5^ New ESC Guidelines recommend short-term ECG recording for at least the first 24 h and continuous ECG monitoring for at least 72 h whenever possible in cryptogenic stroke.^1^

## Methods

We conducted a retrospective study in a single tertiary center in patients who suffered an ischaemic stroke or TIA and performed Holter monitoring between October 2009 and October 2011. All consecutive patients were selected, and those with AF or previous AF were excluded. We followed these patients for 8 to 10 years, observed the incidence of AF and evaluated the clinical, electrocardiographic and echocardiographic predictors of new-onset AF.

Excessive supraventricular ectopic activity (ESVEA) was defined as ≥ 500 premature atrial contractions per 24 hours or any sustained supraventricular tachycardia episode.^6^

Statistical analysis was performed in IBM SPSS Statistics version 25. Categorical variables were compared using the chi-square test, and differences were considered statistically significant when the p-value < 0.05.

## Results

In total, 104 patients were included; 79.5% had a stroke, and 20.5% had a TIA; 45.7% were female; the mean age was 63.8 ± 14.7-year-old at the time of the event (table 1). Concerning cardiovascular risk factors, 59.0% had hypertension, 47.4% had dyslipidemia, 19.5% had diabetes, 43.6% were smokers or previous smokers, and 66.7% were high alcohol consumers. Regarding echocardiographic features, 98% of patients had normal systolic ejection fraction, and only 2% had mildly impaired ejection fraction; medium left anteroposterior atrial diameter was 39 mm and 60% of patients had non-significative mitral regurgitation. 24-hour Holter monitoring revealed ESVEA in 13.5% of patients and paroxysmal AF in 1.9%. All patients with paroxysmal AF detected in Holter monitoring had a stroke and were older than 55.

**Table 1:** Baseline characteristics and Follow-up results

N		104
Age, years (IQR)		63.8 (49.1-78.5)
Female, %		45.7
Hypertension, %		59.0
Dyslipidemia, %		47.4
Diabetes, %		19.5
Smoker or previous smoker, %		43.6
High alcohol consumers, %		66.7
Medium left atrial diameter, mm		39
Left systolic ventricular function, %		
	• Normal	98.0
	• Mildly reduced	2.0
Acute event, %		
	• Stroke	79.5
	• TIA	20.5
Holter results at baseline		
	• AF, %	1.9
	• ESVEA, %	13.5
At follow-up		
	• AF, %	11.5

TIA: transient ischaemic attack; AF: atrial fibrillation; ESVEA: excessive supraventricular ectopic activity.

At a follow-up of 8-10 years, new-onset AF was detected in 11.5% of patients; these had similar mortality compared to those in sustained sinus rhythm (16.7% vs. 21.1%, p=0.724). Alcohol intake, an established risk factor for the development of AF, was associated with a non-significant increase in AF (18.0% vs. 11.5%, p=0.464), while cardiovascular risk factors, left atrium enlargement, mitral regurgitation was not associated with AF development. Regarding premature atrial contractions (PACs), documentation of ESVEA at presentation showed to be significatively associated with new-onset of AF at follow-up (35.7% vs. 8.1%, p=0.003). ESVEA also seems to be related to higher mortality at a long-term follow-up, although this difference was not statistically significant (35.7% vs. 18.6%, p=0.145) (Figure 1).

**Figure 1:** Differences in follow-up between patients with and without ESVEA. ESVEA at presentation was significatively associated with new-onset AF at follow-up (35.7% vs. 8.1%, p=0.003) and seemed to be related to higher mortality (35.7% vs. 18.6% p=0.145). ESVEA: Excessive supraventricular ectopic activit ; AF: atrial fibrillation.

## Conclusion

Our study corroborates previous reports suggesting that excessive PACs increased the risk of death and AF.^6,8^ Copenhagen Holter Study showed that excessive PACs were associated with an increased risk of death, stroke, and admissions for AF at a median follow-up 6.3 years.^7^ The same cohort was followed for 15 years, and the patients with ≥ 30 PACs/hour or with any runs of ≥ 20 PACs had an increased risk of ischemic stroke beyond developing AF. In that study, stroke was often the first clinical presentation than AF.^8^ All these reports and the present study released whether patients with ESVEA benefit from anticoagulation. In fact, the assignment of atrial fibrillation as the cause of stroke totally changes patient’s therapy, so it is of major importance to identify patients with paroxysmal AF and recognize which ones have more risk of having occult episodes of AF. 24-hour Holter monitoring allows the detection of paroxysmal AF, but apparently with low effectiveness. In our study, only 1.9% of patients were identified with AF promptly after stroke or TIA, while 11.5% exhibited AF in long-term follow-up.

Our study showed that ESVEA is a strong predictor of new-onset AF, highlighting the importance of ECG monitoring. This finding, combined with other risk factors such as embolic stroke of unknown source, can be used to identify patients at higher risk of developing AF who benefit from a long-term ECG monitoring or a more regular follow-up.
